# Non-Targeted Metabolite Profiles and Sensory Properties Elucidate Commonalities and Differences of Wines Made with the Same Variety but Different Cultivar Clones

**DOI:** 10.3390/metabo10060220

**Published:** 2020-05-28

**Authors:** Álvaro Cuadros-Inostroza, Claudio Verdugo-Alegría, Lothar Willmitzer, Yerko Moreno-Simunovic, José G. Vallarino

**Affiliations:** 1 metaSysX GmbH, 14476 Potsdam-Golm, Germany; Inostroza@metasysx.com; 2 Centro Tecnológico de la Vid y el Vino, Universidad de Talca, Av. Lircay s/n, 3460000 Talca, Maule, Chile; cverdugo@utalca.cl; 3 Max-Planck-Institut für Molekulare Pflanzenphysiologie, 14476 Potsdam-Golm, Germany; willmitzer@mpimp-golm.mpg.de

**Keywords:** metabolomics, wine, clones, *Vitis vinifera*, sensory analysis

## Abstract

Grapes, one of the oldest agricultural crops, are cultivated to produce table fruits, dried fruits, juice, and wine. Grapevine variety is composed of clones that share common morphological traits. However, they can differ in minor genetic mutations which often result in not only notorious morphological changes but also in other non-visible sensorial distinctive attributes. In the present work, we identified three *Vitis vinifera* cv. Pinot noir clones grown under identical field conditions that showed different grape cluster types. Here, sensorial analysis together with non-targeted metabolite profiles by Ultra High performance Liquid Chromatography (UPLC) couples to Ultra High Resolution Mass Spectrometry (FT-ICR-MS) of wines elaborated from the three different grape cluster types was studied with the aim of (i) finding sensorial differences among these three types of wines, and, if there were, (ii) determining the molecular features (metabolites) associated with these sensorial attributes by a multivariate statistical approach.

## 1. Introduction

With several thousand varieties, the *Vitis* genus is characterized by high levels of genetic diversity. The *Vitis* international variety catalogue identifies more than 21 thousand names of varieties, including 12.250 for *Vitis vinifera*. However, we have to consider that this number includes also synonyms and homonyms [1]. Moreover, the actual number of vine varieties for *V. vinifera* species is estimated at 6000 [2]. Additionally, vines are one of the most important crops in the world with a total surface area planted reaching 7.4 million hectares (Mha and world-wine market reaching 31.3 bn EUR in 2018. The variety Pinot noir is the 6th most widely planted red grape variety worldwide [3]. This cultivar is particularly well adapted to cool growing areas and can be grown at higher latitudes in comparison to other varieties. In addition, the Pinot noir variety is appreciated by the quality of its fresh and fruity monovarietal wine. 

Wine can be considered a complex product whose quality is attributable to its chemical composition, which is highly influenced by grape variety [4], viticultural practices [5,6], fermentation conditions [7], vineyard geographical location that is related to soil and climate characteristics [8,9,10], technical conditions of wine-making [11], and the quality of the grapes used for its production [12,13]. Determination of grape quality is important as grower payment per ton of fruit. Aroma is one of the most important factors in determining wine character and quality [14]. Another equally important wine quality attribute is mouthfeel. Aroma descriptors include red berries, smoky, herbal, and woody, which represent what a wine smells like; while taste is described by attributes such as astringency, bitter, acidity, and sweetness. These characteristics are the result of complex interactions among volatile and non-volatile compounds. So far, the contributions of these attributes to wine are determined by a tasting panel, who must be rigorous and well trained using an appropriate and standardized vocabulary. 

Metabolomic approaches can take a snapshot of the current biochemical status and are useful to compare varieties and evaluate changes in metabolic pools. In this regard, metabolomics-based approaches can reveal several thousand signals of candidate biomarkers providing high sensitivity and good resolution for wine authentication purposes. Recently, there have been a number of reports utilizing high-throughput techniques focusing on wine or grape metabolomes to discriminate wines from distinct varieties, fermentation conditions, geographical origin, as well as different qualities [15,16,17,18,19,20,21,22,23,24,25,26]. Moreover, most of the current analysis of wine with high-throughput analytical technologies contributes to a volatile profiling using different multivariate techniques [17,18,21,27]. However, very little is reported on non-targeted and non-volatile metabolite profiles as a basis [19,20,22,26,28,29]. Furthermore, to the best of our knowledge, none of the non-volatile biomarkers associated with wine quality (sensory properties) have been targeted to assist or confirm the rating score provided by wine panelists. 

Due to the unstudied phenotypic cluster variation observed in an established clonal-commercial vineyard, the scope of this work is to use the combination of sensorial analysis and non-targeted metabolite profiling by ultra-high-performance liquid chromatography coupled to Ultra-High-Resolution Mass Spectrometry (UPLC-FT-ICR-MS) to determine whether both platforms have the power to distinguish between three red wines made using the different cluster types obtained each from three *V. vinifera* cv. Pinot noir putative-clones grown under identical field conditions. In addition, if this is feasible, we are also interested in identifying molecular features which contribute most to the distinction between the different sensory properties of the three types of red wines assessed by an expert panel. Therefore, the selected molecular features can be used as biomarkers for certification or assessment sensorial analysis. 

## 2. Results

### 2.1. Viticultural Characteristics of Three Pinot Noir Grape Cluster Types

**Figure 1:**
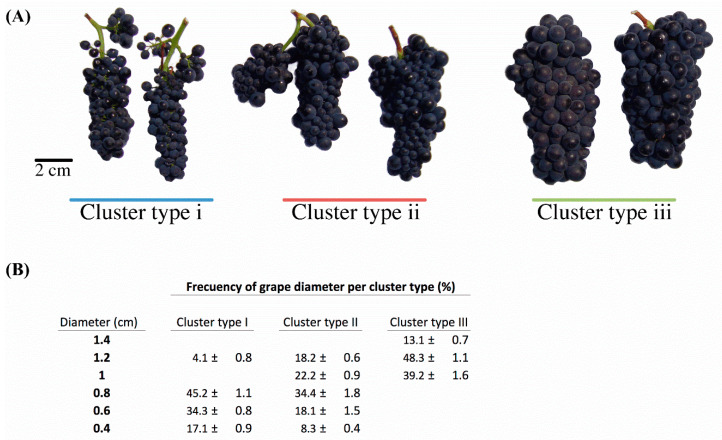
(**A**). A comparison of the three Pinot noir grape cluster types. Scale bar = 2 cm. (**B**). Berry diameter at the ripe stage. Values are mean ± standard deviation (n = 50).

Different fruit size was observed between different cluster types (Figure 1). Grape cluster type iii exhibited a significantly greater berry size in comparison to grape cluster type i, while grape cluster type ii showed an intermediate phenotype, with large, medium, and small grape sizes. Therefore, different ratios between skin and fresh weight were produced by the different types of berries as expected. Although berry size was different, it is apparent that this resulted in an unaltered soluble solids content (Brix), total acidity, and pH of grape berries (Table 1). In wines made from the three grape cluster types, no differences in the alcohol content, residual sugar content, total acidity, and pH were observed. 

### 2.2. Influence of Different Pinot Noir Grape Cluster Types on Sensory Evaluation of Wines

**Figure 2:**
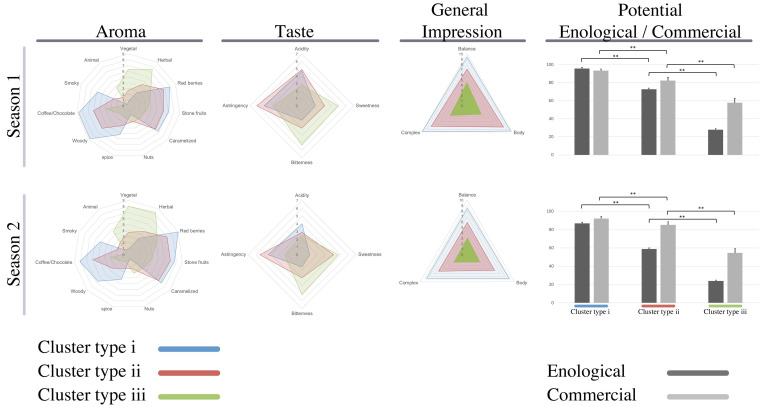
Sensory analysis. Graph of the mean sensory ratings of the three Pinot noir wines studied. Ten experienced Pinot noir wine-makers conducted Duo-Trio test trials to determine the overall difference between wines samples.

A paired comparison test was carried out to evaluate whether trained panelists were able to note organoleptic differences in taste and aroma between wines made from the three grape cluster types. The sensory data were the mean values of all panelists who took part in every season under evaluation. Results showed significant differences among the three classes of wines for all the sensory properties assessed (Figure 2). It is important to note that no significant differences were observed when data from the two seasons were compared. As revealed in Figure 2, the aroma of wines made with grapes from cluster type i was mainly characterized by red berries, stone fruits, caramel, and coffee/chocolate attributes. By contrast, the herbal and vegetal attributes were especially high in the wine elaborated with grape cluster type iii. The other attributes that varied significantly between samples were spice, woody, and smoky which presented high scores in both samples of wine type i and type ii compared with wine made of grape cluster type iii. In addition, we observed that the taste attributes, acidity, and astringency notes were higher in the wines from grape cluster type i and cluster type ii, while sweetness and bitterness attributes were lower. By contrast, in wine obtained from grape cluster type iii, sweetness and bitterness attributes were enhanced by reduced acidity and astringency. Also, when the panelists were asked about their general impression of these wines, again those made with grapes from cluster type i were the best evaluated. In contrast, larger grapes seem to generate wines with less relative balance, body, and complex attributes. Panelists were also asked to score the wines from 0 to 100 points in terms of enological potential to be used in wine blend and also about their commercial value if those wines were bottled and sold as monovarietal wines. As before, those wines made with cluster type i received the best scores. Statistically significant differences were observed between the qualifications of the wines made with all different cluster types. Overall, given these sensory attributes for the three wine types, the panelists classified as the most well-rounded and with better enological/commercial potential the wine made from grape cluster type i.

### 2.3. Untargeted Metabolite Profiles of Three Pinot Noir Wines

**Figure 3:**
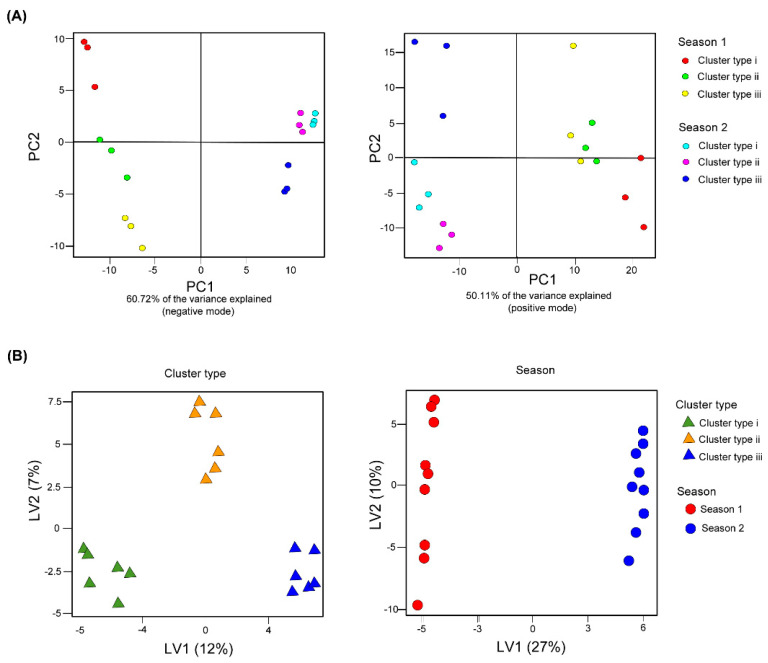
Score plots of all detected *m/z* features of the three Pinot noir wines from two different seasons in positive and negative mode investigated by (**A**) principal component analysis (PCA) and (**B**) the latent variables (LVs) in first discriminant analysis (sPLS-DA).

Given the differences in the sensory analysis of the three types of wines, we were next interested in determining whether the unrestricted metabolomics analysis of these types of wines allowed us to significantly associate non-volatile metabolites with specific aromatic and taste attributes. To this end, an exhaustive untargeted analysis of secondary metabolite profiling was conducted on wines produced from the three grape cluster types. Wines from the two seasons were analysed using a UPLC-FT-ICR-MS in positive and negative ionization mode. Based on visual inspection of the raw total ion chromatograms, the wine samples showed clear differences depending on the grape cluster types (data not shown). The data sets were examined by principal component analysis (PCA) (Figure 3A), with two principal components explaining 50.11% and 60.72% of the overall variance of the detected features in positive and negative mode, respectively. The PCA revealed two distinct patterns. The analysis highlighted a sharp season-dependent trend (PC1), while PC2 showed clear differences that were evident between the three types of wines from the same season. This result strongly indicates that the PCA-derived separation was not based only on the presence/absence of grape cluster type specific features but also on quantitative differences within the metabolites present in all wines.

In light of such results, linear discriminant analysis (sPLS-DA) was used as a supervised classification technique [30] to develop models capable of predicting the classification of wines according to the grape cluster type and harvest year. A plot of the two first discriminant variates (derived from all *m/z* features measured in either positive or negative ionization mode) is presented in Figure 3B. When the discrimination was based on harvest year, a similar pattern was observed compared with the unsupervised PCA (Figure 3A). However, when grape cluster type was considered as a variable, we observed a clear separation into three groups according to the grape cluster type independent of harvest year (Figure 3B).

### 2.4. Multivariate Discrimination of Three Pinot Noir Wines Based on Metabolic Profiling and Identification of Multiple Metabolic Features as Biomarkers of Wine Quality

Since the quality of the three wine types were scored differently based on their sensory evaluation, we next constructed sparse Partial Least Squares (sPLS) regression models in order to identify *m/z* features (biomarkers) related to sensory attributes and that also distinguished different harvest years and cluster types. An sPLS model was generated for each dependent variable (wine attributes), and the normalized intensity levels of the molecular features were used as predictors. The relative importance of each independent variable (*m/z* feature) is measured in terms of the VIP (variable importance in projection) scores; the higher the VIP scores, the greater the association of the analysed *m/z* feature with respect to the dependent variable (sensory attribute). The *m/z* features with the highest VIP score (> 1.0) are displayed in Table S1. The identified molecular features were then subjected to compound annotation.

Annotation of metabolites based on high resolution mass spectrometry accurate mass provides an initial indication of the potential identification. A match to a potential molecular formula, as defined by the metabolomics standards initiative (MSI), corresponds to a level 2-based identification. Level 3 identification is for *m/z* features that match multiple molecular formulas but within a single class of metabolites, while unknown features are classified as level 4 [31]. In order to classify compounds with higher confidence, we also used the MS2 spectral data (unit mass and peak ratio match) for identification. When MS2 spectral data were not captured for a given metabolite, data acquired with previous grape and wine samples analysed under identical conditions were considered, together with published data. Where a given compound ionized in negative and positive modes and different adduct species were observed for each, the MS2 spectra data for each ion species were manually assessed and compared.

We were able to tentatively identify a total of 26 molecular features with a VIP score higher than 1.0 that exhibited a significant contribution to explain the variation between harvest years. In concordance with the results obtained in the sensorial analysis with respect to the lack of discrimination of wines made from the same cluster type but in different seasons, any of those features were in common with the features found to be associated with all measured sensory attributes.

**Figure 4:**
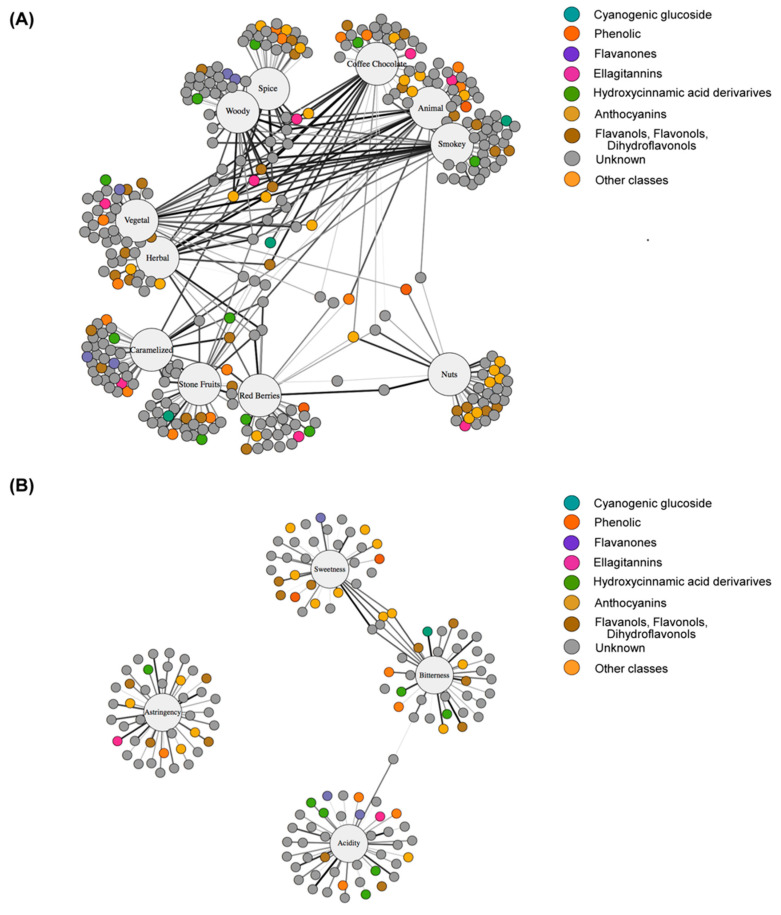
Visualization of sensory attribute-molecular feature correlations in Pinot noir wines. (**A**) Network considering aroma attributes and (**B**) taste attributes. Notes are as follows: sensory attributes are represented as large circles, while molecular features are represented as small circles and are color coded by compound category. The edge thickness and color indicate the strength of the association; darker and thicker lines mean stronger associations (only those that showed a VIP coefficient > 1.0).

To better visualize the molecular features that significantly contribute to specific sensory attributes, we performed an association study using networks (Figure 4, Table S1). In each network, sensory attributes and molecular features are represented as nodes of different size (large and small) and are color coded by compound category. An edge represents an association between nodes if the corresponding VIP coefficient is greater than 1.0. The edge thickness and color indicate the strength of the association; darker and thicker lines mean stronger associations. Network analysis emphasized links both between and within the various sensory attributes. From the analysis for sensory evaluation associated with aroma and taste attributes (a total of 15 sensory attributes were addressed; spice, woody, smoky, coffee/chocolate, vegetal, animal, herbal, red berries, stone fruits, nuts, caramel, astringency, acidity, bitterness, and sweetness), we observed a total of 65 polar *m/z* features that displayed good VIP score as listed in Table S2. Following the identification process, 22 secondary metabolites were tentatively assigned to specific compound categories, eight flavonols and flavanols, eight anthocyanins, one hydroxycinnamic acid derivative, two ellagitannins, two cyanogenic glucosides, two classifieds as phenols, and 43 other classes (Figure 4, Table S2).

Interestingly, the network associated with aroma attributes (Figure 4A) revealed markedly higher connectivity than the network for taste attributes (Figure 4B), suggesting that aroma attributes in wine may represent a higher level of complexity as compared with taste attributes. Comparative network analysis also suggested that molecular features associated with taste attributes were largely modular, while features associated with aroma attributes showed more complex and interconnected associations between specific features. The aroma attributes network analysis revealed five interesting clusters, one of which included red berries, stone fruit, and caramel attributes, another involved spice and woody characteristics, while two other clusters contained herbaceous characters (herbal and vegetal) and coffee/chocolate, smoky, and animal, respectively. Interestingly, we observed a strong link between nut attributes and the two aroma attributes, coffee/chocolate and red berries. For network involving taste attributes, we observed specific *m/z* features strongly associated with the four tested taste attributes. Here, we observed an association between sweetness and bitterness attributes, while acidity and astringency displayed non-clear association with the other analysed attributes. Interestingly, the link between sweetness and bitterness attributes was through four *m/z* features; two of them were annotated as anthocyanins (named catechylpyranopeonidin-3-O-glucose and pyranopeonidin 3-O-coumaroyl-glucose), although in general, Pinot noir wines have been described to have lower anthocyanin concentrations [32].

## 3. Discussion

The quality of a wine is known to directly correlate with the quality of the grapes used for its production [13]. Therefore, quality and character of a wine should correlate directly with the chemical composition of grape [33].

The starting point of this study was due to an interesting observation made by the grower in an established vineyard, in which a Pinot noir clone grown under the same mesoclimate and treated using the same viticultural practices produced three different grape cluster types (Figure 1).

High-throughput metabolomics facilitates dissecting a phenotype at the metabolite level, potentially allowing a more holistic perspective in monitoring and gaining information on the winemaking process, and thus, this approach can assist in the evaluation of sensorial attributes and can potentially help the improvement of several aspects of wine quality.

The first aim of the present study was to test if a comparative metabolome and sensorial analyses of three Pinot noir wines made by three different grape cluster types were able to discriminate wine types. If this was feasible, we were also interested in better understanding the diversity in the sensory attributes of these wines. For that, we tested a multivariate statistical method to identify metabolites or molecular patterns (biomarkers) related to different sensory attributes, which can be used to assist the sensory analysis postulated by wine expert panelists.

The wine made from grape cluster type iii revealed significant organoleptic differences from the other two wine types. In particular, we observed that wine produced by grape cluster type iii was found to have greater scores for vegetal and herbal aromas. On the other hand, wine types i and ii showed differences by displaying more canned fruity characteristics (red berries, stone fruits, and caramel). Moreover, wine type i was the most intense in woody and coffee/chocolate characters. This strong discrimination between herbaceous and fruity attributes within wines produced from the same grape variety has been previously reported [34,35,36,37]. In these studies, the authors also indicated that the descriptive profile of Cabernet Sauvignon wines displayed a dichotomy between herbaceous and fruity characteristics.

With respect to the taste attributes, wine obtained from grape cluster iii was classified as having a reduced mouthfeel in terms of astringency and acidity. It was also noted that wine produced from grape cluster type iii had a noticeable increase in bitterness and sweetness. High levels of organic acids have been associated with an increased perception of astringency. Furthermore, this perception is also pH dependent [38]. Here, although we did not observe significant differences in total acidity and pH, we detected differences in the level of malic acid in wine made from grape cluster type iii compared to the other types. Moreover, astringency of high-molecular-weight polyphenolics (tannins) can be enhanced by the presence of organic acids [38,39]. Therefore, this observation agrees with the hypothesis that different concentrations of tannins in wine type iii can contribute to the observed differences in astringency. Additionally, we observed increased ethanol levels in wine types i and ii, although non-significant, which can have a greater effect on perceived astringency than the difference between acid and high-molecular-weight polyphenol levels [40]. Similarly, a high ethanol level is described to increase the perception of bitterness [41,42,43]. However, we observed that bitterness was perceived to be higher in wine type iii, although previous studies suggest that the bitterness perception can be suppressed by the sweetness [41]. However, we observed that wine type iii was classified with higher values for sweetness and bitterness attributes, suggesting that these sensory attributes cannot reflect the same chemical properties when comparing different cultivars. Consistent with this hypothesis, we observed no significant changes in residual sugar levels when comparing the three wine types, although it has been described that levels of sugars in wine correlate with the sweetness perception [44].

In view of the fact that wine is a really complex matrix and the levels of individual metabolites are not necessarily as important as their interaction with other compounds, the use of metabolomics allows a holistic perspective in monitoring several aspects of wine quality. Metabolomics have been successfully applied to discriminate and classify wines according to grape variety, geographic origin, age or winemaking practice [15,16,17,18,19,20,21,22,23,24,25,26]. Therefore, we decided to further use mass-spectrometry metabolomics to investigate if we could establish an association between non-volatile metabolites or features and specific sensory attributes related to wine quality.

For this purpose, wines produced by the three grape cluster types and classified differently according to their quality by wine experts were analysed using UPLC-FT-ICR-MS. This analysis revealed that the different wine types underwent several changes in secondary metabolite concentration, mainly phenolic compounds. We focused on these classes of metabolites since they are important chemical components of wines and can strongly influence the final organoleptic perception [45,46,47]. The content and presence of these classes of metabolites alone enabled us to discriminate between years as demonstrated by PCA, in which the two analysed seasons can be clearly distinguished, in agreement with previously published studies on other classes of wines [19]. In addition, this analysis was also able to classify wines according to the grape cluster types used for winemaking as shown in the sPLS-DA model (see Figure 3B). In this study, we have described that the putative biomarkers discriminating the wine quality are related to changes in their abundance. Therefore, our results reveal that our metabolite profiling provided sufficient discriminatory power to differentiate wine according to its quality. We were then interested in identifying potential biomarkers associated with sensory wine attributes as scored by a panel of experts. To that end, we applied sparse Partial Least Squares (sPLS) regression models using the experts’ scores as dependent variables. From the metabolite-attribute networks constructed from the sPLS models, we observed that the network associated with aroma attributes revealed markedly higher connectivity than the network for taste attributes, suggesting that aroma attributes in wine may represent a higher level of complexity as compared with taste attributes (Figure 4). After that, we focused our attention on the molecular features that were strongly associated with specific sensory attributes (VIP > 10) to assess whether we were able to putatively annotate them.

We found that specific anthocyanins were strongly associated with different aroma attributes (Figure 4 and Table S1). These pigments can be grouped as glycosides (i.e., monoglucosylated anthocyanins), acylated pigments (i.e., acylated anthocyanin monoglucosides, in which the acylated group can be *p*-coumaric acid, caffeic acid or acetic acid), and anthocyanin-derived pigments (i.e., pyranoanthocyanins and tannin-anthocyanin dimers). The Pinot noir anthocyanin profile is unique as it lacks (except in traces) acylated anthocyanins [48,49]. In this study, we found pyranopeonidin-3-O-glc to be associated with caramel, vegetal, herbal, coffee/chocolate, spice, woody, and smoky attributes, while catechylpyranopeonidin-3-O-glc was associated only with the nut character. It has been described that these chemical compounds are produced in wines during fermentation and the aging process [50,51]. Furthermore, two other studies have demonstrated that pyranoanthocyanins can also be found in red grapes postharvest [52,53]. In addition to these anthocyanin compounds, we identified pelargonidin 3-O-glc to be strongly associated with the animal attribute (VIP = 28.603). Interestingly, this anthocyanin has been detected at a very low concentration in comparison to other anthocyanins in red and pink grape berries [54]. This finding can suggest that pelargonidin 3-O-glc is a clear candidate to be a chemical marker associated with the animal attribute in Pinot noir wines. In most f *V. vinifera* varieties, anthocyanins are known to be present only in the skin of red grape cultivars and together with the co-pigments or anthocyanins-related pigments are responsible fof the red colour shown by red wine [55]. Anthocyanins are odorless molecules which can conflict our result. However, anthocyanins are known to modulate the formation of polymeric pigments [56] which are adducts formed during winemaking that provide mouthfeel properties [57]. In addition, anthocyanin-derived pigments have precursors molecules of different chemistries, including vinyl- and ethyl-phenols [58] or tannins [59], and we cannot discard their influence on aroma.

Resveratrol is a non-flavonoid compound in the stilbenoid class. In particular, the biomedical literature focuses attention on *trans*-resveratrol (3,4,5’trihydroxy-*trans*-stilbene) because it shows a strong antioxidant capacity and it has been positively associated with health effects, for instance, in cardiovascular disease and colon and breast cancers [60,61]. In wine, this compound has been found to be present around 10 times higher in red varieties than in white varieties [62]. Piceid, the β-D-glucoside form of resveratrol (resveratrol 3-O-β-D-glucoside), is also found in wines [63] and has been physiologically described as important as *trans*-resveratrol [64,65]. Interestingly, our data revealed that the piceid level in wine was associated with coffee/chocolate. We are unaware of any previous research that showed how the piceid level influenced wine quality; however, it has been described that a high level of *trans*-resveratrol did not alter the flavor or aroma profile of Cabernet Sauvignon [66].

Following the same strategy to identify chemical compounds associated with mouthfeel attributes, we focused our interest on those features with a VIP score > 10. We observed that the sPLS model associated with taste was less dense that the model from aroma characters. The model clearly showed that the compounds responsible for the discrimination between the four tested characters (sweetness, bitterness, acidity, and astringency), at least from the putative annotated features, are mainly represented by anthocyanins, flavanols, and flavanols species (Figure 4). It is well understood that monomeric flavan-3-ols and oligomeric tannins, particularly catechin and epicathechin species, are associated with astringency perception of red wines [41,67,68]. Interestingly, we identified catechin (VIP = 17.19) that correlated with the astringency attribute. Therefore, we can suggest that the level of catechin can act as a chemical marker for the astringency attribute in Pinot noir wines. However, the level of variation of this metabolite that can be associated with this sensory character will need further investigation.

From this study, we can demonstrate that wine quality in term of sensory analysis is a complex process in which the individual level of volatile and non-volatile compounds but also their interaction play an important role. Here, we presented a metabolic pipeline methodology allowing

(i) efficient discrimination of different wine quality
(ii) identification of *m/z* features associated with specific sensory attributes.

However, further research on the validation of the identified *m/z* features (putative annotated and non-annotated) related to aroma and taste characters is needed to release metabolic biomarkers to assist panelists with wine quality discrimination.

## 4. Materials and Methods

### 4.1. Chemicals

All solvents were of High Performance Liquid Chromatography (HPLC) grade and were obtained from VWR International. Other chemicals were of the highest purity grade available and were obtained from Sigma–Aldrich. Water was of Milli-Q quality (Millipore Corp., Bedford, MA, USA).

### 4.2. Experimental Design

An established commercial vineyard located in Casablanca valley, Chile (33°18′59″ S 71°27′30″ W) was used in this study. The grapevines were clonally multiplied, and the field was established seven years before this study. The experiments were performed on *V. vinifera* cv. Pinot noir during the 2008/2009 and 2009/2010 seasons. Vine spacing was 1.2 m between vines and 2.2 m between rows. Vine management was performed by the grower according to the regional appropriate viticultural practices for this *V. vinifera* cultivar. The vines were cane pruned for a vertical shoot-positioning trellis system. No rain occurred after fruit set. Irrigation was applied to vines in order to maintain midday leaf water potential between −0.6 and −0.8 MPa throughout the season. Considering the phenotypic cluster difference among plants in the field, each experimental unit consisted of a few interior vineyard panels in a randomized complete block design. Individual grapevine plants and cluster samples were selected at random from throughout a vineyard block at the ripe stage.

### 4.3. Wine Samples

Clusters from the different selected blocks were used to make microvinifications. Wines were made from each selection group according to standard winemaking practices (Centro Tecnológico de la Vid y el Vino, Universidad de Talca) with four biological and three technical replicates. Each replicate consisted of 100 ± 1 kg of hand-harvested fruit collected from the field-selected blocks. Grapes were destemmed, and the berries were inoculated with yeast (*Saccharomyces bayanus*, EC-1118, Lallemand, 1620 Rue Préfontaine, Montréal, QC H1W 2N8, Canada) at a rate of 20 g hL^−1^ in stainless steel fermenters. Cap management was performed twice per day by manual punch-downs. The fermentation temperature was maintained between 22 °C and 23 °C at a density of 990 g/L. After alcoholic fermentation, wines were pressed and racked into carboys and inoculated with Enoferm Alpha (Lallemand, 1620 Rue Préfontaine, Montréal, QC H1W 2N8, Canada) to start malolactic fermentation. At the end of malolactic fermentation, K_2_S_2_O_5_ was added to 30 ppm of SO_2_ in the wine. In addition, a cold stabilization at 2 °C was performed before bottling. Bottling and capping were performed manually.

### 4.4. Wine Sensory Analysis

Duo-Trio test trials were conducted to determine the overall difference between wines samples. Ten experienced Pinot noir wine-makers, highly trained and experienced as Pinot noir enologists, formed the wine panelists (four females and six males between the ages of 30 and 58). To minimize carry-over of flavors, the panelists were asked to rinse their mouth with water between samples, and the test was repeated on different days. One month after bottling, the wines were evaluated.

### 4.5. Metabolite Measurements

Chromatographic separation, mass spectrometric measurements, and data analysis were performed using a Waters Acquity Ultra High Resolution Liquid Chromatography (UPLC) system (Waters, Milford, MA, USA) using an HSS T3 C_18_ reverse phase column (100 × 2.1 mm i.d., 1.8 µm particle size; Waters) which operated at a temperature of 40 °C. The mobile phases consisted of 0.1% formic acid in water (Solvent A) and 0.1% formic acid in acetonitrile (Solvent B). The flow rate of the mobile phase was 400 µL/min, and 2 µL of sample was loaded per injection. The gradient was: 0–1 min isocratic flow at 99% A, 1–12 min linear gradient from 95% to 65% A, 12–13.5 min linear gradient from 65% to 20% A, 13.5–14.5 min linear gradient from 20% to 1% A, 14.5–16 min isocratic flow at 1% A, 16–17 min linear gradient from 1% to 95% A, and 17 to 20 min isocratic flow at 95% A, to re- equilibrate the column before to the next sample could be injected. The UPLC was connected to an Ultra High Resolution Mass Spectrometry (FT-ICR) via a TriVersa NanoMate (Advion, Ithaca, NY, USA). The spectra were recorded alternating between full-scan and all ion-fragmentation-scan modes, covering a mass range from 100 to 1500 *m/z* using the LTQ FT-ICR-Ultra Mass Spectrometer (Thermo-Fisher, Bremen, Germany). The resolution was set to 35,000, and the maximum scan time was set to 250 ms. The sheath gas was set to a value of 50, while the auxiliary gas was set to 20. The transfer capillary temperature was set to 250 °C, while the heater temperature was adjusted to 350 °C. MS spectra were recorded from minute 0 to 19 of the UPLC gradient. All samples were randomized prior to mass spectrometric analyses to avoid any experimental drifts. A number of internal standards (chloramphenicol, corticosterone, and ampicillin), added to each sample just prior to injection, were used to control experimental variability. One month after bottling, the wines were chemically evaluated.

### 4.6. Metabolite Annotation and Statistical Analysis

Pre-processing of raw chromatograms was performed using Expressionist Refiner MS 10.0 (GeneData; http://www.genedata.com) with an established workflow. This workflow included baseline correction, removal of chemical noise, and chromatogram alignment. As output, a list of molecular features, that is, a retention time and a *m/z* ratio pair, and a data matrix containing relative intensities for each feature and for each chromatogram were obtained. All further computations, data manipulations, and plot generation were carried out using the R programming language (http://www.r-project.org).

The data matrix was further filtered for low intense features whose mean intensity across all samples was less than 0.1% of the highest average. Moreover, features with more than 30% missing values were also removed as well as features that eluted before 0.45 min. After filtering, the *m/z* feature data were normalized by sample volume and sample median intensity. Due to machine sensitivity variation across different measurement runs, each feature was normalized by dividing its intensity in a sample by the median intensity across all measurements within a batch in order to compensate for said effect.

Principal component analysis (PCA) was performed in R using the package pcaMethods [69] using five components and unit variance scaling. Sparse partial least squares (sPLS) and Sparse partial least squares discriminant analysis (sPLS-DA) were performed using the package mixOmics [70]. The sPLS-DA was used for categorical variables (season and cluster type) while the sPLS was used for quantitative data (sensorial attributes). In both cases, the metabolite levels were considered predictors. For each response variable (categorical or quantitative), a single sPLS/sPLS-DA model was established. To determine the optimal number of components and variables of a given model, we searched the parameter space spanned by 1 to 12 (3 for sPLS-DA) components and 50, 100, 200, 300, 500, 1000 selected variables. For each such component/variable combination, 100 iterations of 5-fold cross-validation rounds were tested and the pair that resulted in the lowest (sPLS-DA) or highest R2 (sPLS) classification error was taken as the optimal parameters. Once an optimal number of components and variables was determined for each response variable, we computed the respective sPLS/sPLS-DA model and using this, we obtained the variable importance in projection (VIP) coefficient for each metabolite. Finally, we used the VIP coefficients to generate networks in which a node represents either a molecular feature or a sensorial parameter, and an edge was drawn if the respective VIP coefficient was greater than zero. All networks manipulations were performed using the igraph package [71].

Molecular features were putatively annotated by searching the *m/z* value against the KEGG compound database. A maximum tolerance of 5 ppm was allowed, considering the following potential known adducts: M+H, M+Na, M+NH_4_ for positive mode and M-H, M+AcOH-H for negative mode. A custom Python script was written for this task. The MS/MS fragmentation of the metabolites was compared with candidate molecules found in databases and verified with earlier literature on similar compounds, especially when the presence of the metabolite was reported in grapes and wines.

## Figures and Tables

**Table 1:** Grape and wine analysed in the experiment including some basic compositional parameters. Values are mean ± standard deviation (n = 50 and n = 6 for grapes and wines, respectivately.

Season	Cluster Type	Grapes				Wines			
			Average	±	SD		Average	±	SD
**Season 1**	Type i	ºBrix	24.8	±	2.92	Alcohol (% vol.)	14.4	±	0.31
						Residual sugar (gr/L)	1.3	±	0.17
		Total Acidity (HSO_4_ gr/L)	7.5	±	1.11	Total Acidity (HSO_4_ gr/L)	4.3	±	0.27
		pH	3.0	±	0.11	pH	3.4	±	0.01
		Malic acid (mg/L)	3455.2	±	78.82	Malic acid (mg/L)	1352.1	±	45.89
		Tartaric acid (mg/L)	5471.1	±	61.23	Tartaric acid (mg/L)	2622.2	±	53.21
		Total phenolics (mg GAE)	539.7	±	33.31	Total phenolics (mg GAE)	1825.1	±	23.44
	Type ii	ºBrix	23.7	±	0.38	Alcohol (% vol.)	13.9	±	0.12
						Residual sugar (gr/L)	1.3	±	0.31
		Total Acidity (HSO_4_ gr/L)	6.9	±	0.38	Total Acidity (HSO_4_ gr/L)	4.1	±	0.08
		pH	3.1	±	0.04	pH	3.4	±	0.04
		Malic acid (mg/L)	3322.4	±	86.71	Malic acid (mg/L)	1423.8	±	54.98
		Tartaric acid (mg/L)	5480.5	±	33.11	Tartaric acid (mg/L)	2679.1	±	57.34
		Total phenolics (mg GAE)	576.1	±	47.14	Total phenolics (mg GAE)	1870.1	±	42.32
	Type iii	ºBrix	20.9	±	1.73	Alcohol (% vol.)	12.6	±	0.26
						Residual sugar (gr/L)	1.4	±	0.15
		Total Acidity (HSO_4_ gr/L)	7.5	±	0.99	Total Acidity (HSO_4_ gr/L)	3.7	±	0.33
		pH	3.1	±	0.09	pH	3.4	±	0.12
		Malic acid (mg/L)	3089.3	±	67.32	Malic acid (mg/L)	1200.6	±	66.67
		Tartaric acid (mg/L)	5190.2	±	45.33	Tartaric acid (mg/L)	2499.2	±	71.10
		Total phenolics (mg GAE)	557.9	±	52.41	Total phenolics (mg GAE)	1735.2	±	33.42
**Season 2**	Type i	ºBrix	23.0	±	0.67	Alcohol (% vol.)	13.5	±	0.89
						Residual sugar (gr/L)	1.3	±	0.10
		Total Acidity (HSO_4_ gr/L)	5.5	±	0.82	Total Acidity (HSO_4_ gr/L)	4.4	±	0.27
		pH	3.2	±	0.11	pH	3.4	±	0.12
		Malic acid (mg/L)	3333.3	±	66.62	Malic acid (mg/L)	1487.1	±	87.92
		Tartaric acid (mg/L)	5379.3	±	58.32	Tartaric acid (mg/L)	2598.5	±	58.72
		Total phenolics (mg GAE)	522.1	±	31.23	Total phenolics (mg GAE)	1799.4	±	35.09
	Type ii	ºBrix	24.0	±	1.07	Alcohol (% vol.)	13.1	±	0.65
						Residual sugar (gr/L)	1.3	±	0.25
		Total Acidity (HSO_4_ gr/L)	6.0	±	1.01	Total Acidity (HSO_4_ gr/L)	4.2	±	0.08
		pH	3.1	±	0.15	pH	3.4	±	0.04
		Malic acid (mg/L)	3322.3	±	88.10	Malic acid (mg/L)	1376.2	±	88.78
		Tartaric acid (mg/L)	5380.2	±	30.22	Tartaric acid (mg/L)	2683.9	±	66.91
		Total phenolics (mg GAE)	518.9	±	48.80	Total phenolics (mg GAE)	1754.3	±	55.42
	Type iii	ºBrix	23.7	±	0.77	Alcohol (% vol.)	12.3	±	0.35
						Residual sugar (gr/L)	1.3	±	0.15
		Total Acidity (HSO_4_ gr/L)	5.5	±	0.30	Total Acidity (HSO_4_ gr/L)	3.8	±	0.22
		pH	3.2	±	0.12	pH	3.3	±	0.09
		Malic acid (mg/L)	3101.3	±	65.31	Malic acid (mg/L)	1246.8	±	86.34
		Tartaric acid (mg/L)	5060.2	±	82.23	Tartaric acid (mg/L)	2501.2	±	79.98
		Total phenolics (mg GAE)	513.1	±	50.22	Total phenolics (mg GAE)	1765.2	±	67.72

## Supplementary Materials

The following are available online at https://www.mdpi.com/2218-1989/10/6/220/s1, Table S1. Table of all features associated with specific attributes (VIP score > 1.0). Table S2. Identification of all molecular features that significantly contribute to specific sensory attributes (VIP score > 1.0).
